# Use of the Internet for Health Information by the Chronically Ill

**Published:** 2004-09-15

**Authors:** Todd H. Wagner, Laurence C. Baker, M. Kate Bundorf, Sara Singer

**Affiliations:** Veterans Affairs Palo Alto Health Care System, Palo Alto, Calif, Department of Health Research and Policy, Stanford University School of Medicine, Stanford, Calif, Center for Health Policy and Center for Primary Care and Outcomes Research, Stanford University, Stanford, Calif; Department of Health Research and Policy, Stanford University School of Medicine, Stanford, Calif, Center for Health Policy and Center for Primary Care and Outcomes Research, Stanford University, Stanford, Calif; Department of Health Research and Policy, Stanford University School of Medicine, Stanford, Calif, Center for Health Policy and Center for Primary Care and Outcomes Research, Stanford University, Stanford, Calif; Center for Health Policy and Center for Primary Care and Outcomes Research, Stanford University, Stanford, Calif, Harvard School of Business, Cambridge, Mass

## Abstract

**Introduction:**

Chronic conditions are among the leading causes of death and disability in the United States. The Internet is a source of health information and advice for individuals with chronic conditions and shows promise for helping individuals manage their conditions and improve their quality of life.

**Methods:**

We assessed Internet use for health information by people who had one or more of five common chronic conditions. We conducted a national survey of adults aged 21 and older, then analyzed data from 1980 respondents who had Internet access and who reported that they had hypertension, diabetes, cancer, heart problems, and/or depression.

**Results:**

Adjusted rates for any Internet use for health information ranged from 33.8% (heart problems only) to 52.0% (diabetes only). A sizable minority of respondents — particularly individuals with diabetes — reported that the Internet helped them to manage their condition themselves, and 7.9% said information on the Internet led them to seek care from a different doctor.

**Conclusion:**

Use of the Internet for health information by chronically ill patients is moderate. Self-reported effects on choice of treatment or provider are small but noteworthy.

## Introduction

Chronic conditions are among the leading causes of death and disability in the United States (1) and are responsible for a disproportionately large share of health care use and cost (2). New technologies frequently target people with chronic conditions with the hope of increasing system efficiencies and improving patient quality of life. One particularly promising area of innovation has been consumer-oriented health information on the Internet.

Certain attributes of the Internet make it particularly appealing for patients with chronic illnesses. The cost of distributing information on the Web is extremely low, and people in rural areas and those with disabilities can access the same information as people in urban areas and those with no disabilities. Also, compared with printed documents, Internet information can be easily updated to reflect new scientific findings. This has become particularly useful for patients looking for cutting-edge treatments and new clinical trials for chronic illnesses such as human immunodeficiency virus (HIV) and cancer. While the first few generations of Internet sites offering health information consisted primarily of digitized copies of printed materials, developers were quick to exploit the Internet's interactive capabilities. Consumers can now use the Internet to search for risk-assessment tools, interactive health advice, and the latest medical news.

Researchers have begun to examine the potential effects of the Internet on health issues. The low cost of distributing information on the Internet has prompted some researchers to test whether the Internet could be used as a disease-management tool. In two studies, high-quality, disease-specific information was distributed to randomly selected participants, and participants in control groups received information in the traditional form of a health magazine or book (3,4). Study results indicated that the Internet was a better conduit for providing health information. It is not clear, however, which characteristics of the Internet drive its effect on patient education. One study compared a group that received tailored information and personalized feedback via the Internet with a group that received Internet information only and found few differences between the two groups (5).

In addition to disease management, two other areas of Internet health information research have attracted attention. First, studies by Jadad et al (6), Eysenbach et al (7), and Berland et al (8), among others, have shown that the Internet is saturated with both good and bad health information and that consumers are not good judges of quality. Second, studies have described how people are using the Internet for health information. Although the estimates of how many people use the Internet for health have been heavily debated (9-12), questions about how a person's health affects their use of the Internet have not been investigated in as much depth. A recurring finding is that people with depression are more likely than people with other health conditions to use computers to find health information. This finding was noted as part of a community-wide intervention that provided participants with self-care books, a telephone advice line, and computerized health information (13). Compared with participants without depression, participants with depression reported a higher probability of using all three media. More recently, Haviland and colleagues analyzed data from the 2001 Healthcare Market Guide survey and found that people who reported a psychiatric condition (including depression) were more likely to use the Internet to access disease/wellness information than people with no chronic health problem (14). Similarly, a recent national survey by the Pew Internet and American Life Project found that depression, anxiety, or mental-health issues were among the 10 most frequent health-related search topics on the Internet (15). But beyond depression, there is little research from which to identify other emerging themes.

People with chronic conditions have unique needs; this paper investigates Internet use among respondents who reported having hypertension, diabetes, cancer, heart problems, and/or depression. We conducted a nationally representative survey to assess Internet use for health advice and information by individuals with these chronic conditions. Our analysis focused on individuals with at least one of five common chronic illnesses: diabetes, hypertension, cancer, heart problems, and depression. This study addressed three issues. First, we assessed the extent to which people with any of the five chronic illnesses used the Internet for health information. Second, we compared perceptions of the Internet among participants who have one of the five chronic illnesses. Third, we examined respondents' self-reported effects of the information. The sample used in this analysis represents a sub-population of a previously published paper (9).

## Methods

### Survey of health and the Internet

We surveyed a sample representing the entire U.S. population aged 21 years and older. We drew our sample from a research panel of more than 60,000 households; the panel was developed and maintained by Knowledge Networks (KN), a survey research firm. Using random-digit dialing, KN contacted potential panel households, offering them free Internet access in exchange for periodic participation in short surveys. Participants were informed of their rights as panel members, including the right not to answer surveys or questions. We then surveyed a random sample of panel members through the MSN WebTV.

The electronic survey was sent to a specific household member. A light on the WebTV notified potential participants about the survey. KN formatted the survey for the WebTV to resemble other surveys it sends to panel members. Item nonresponse for variables analyzed in this paper was less than 2%.

Institutional Review Boards at Stanford University and Research Triangle Institute approved the survey protocol. KN sent a consent form and the survey to a sample of 12,878 panel members in late 2001 and early 2002. Those who did not respond within three days received an e-mail reminder. Two additional e-mail reminders were sent to nonrespondents. Of the 12,878 persons who were sent a survey, 2265 (18%) declined consent, 1678 (13%) did not complete the consent form, and 8935 (69%) provided informed consent and subsequently completed the survey. Compared with respondents, people who did not complete the survey were younger on average (49 versus 54 years; *P* < .001) and more likely to have a high school education or less (39% versus 49%; *P* < .001).

A focus of the survey was to assess how people with chronic illnesses were using the Internet. In the survey, we asked people about five common chronic conditions: hypertension, diabetes, cancer, heart problems, and depression. We selected these conditions based on their prevalence and on research by Berland et al (8). Of the 8935 respondents, 4990 reported one or more of these conditions.

We further narrowed our analytical sample because KN had given Internet access to most of our respondents for the first time, and previously published studies on health and the Internet had sampled only people who had obtained Internet access on their own. Thus, of the 4990 respondents, we analyzed data from only the 1980 respondents who had Internet access prior to participation in KN. In a separate paper, we reported on how all people with Internet access prior to participation in KN used the Internet for health (9). In this paper, we focus on people with chronic conditions.

At the time of our survey, the panel recruitment response rate was 41%, calculated by standards established by the American Association for Public Opinion Research (16), and the panel attrition rate was 14%. We independently investigated the generalizability of the KN dataset by comparing disease prevalence estimates to the 2000 National Health Interview Survey (NHIS). The questions on cancer were most similar, and the prevalence estimates were 6.2% for our sample and 6.8% for NHIS. For diabetes and hypertension, our questions differed from the NHIS questions: we asked about diabetes along with high blood sugar and hypertension along with high blood pressure, rather than just about diabetes and hypertension. Prevalence estimates in our sample were 12.3% for diabetes and 29.0% for hypertension — each approximately five percentage points higher than the NHIS survey. We also conducted additional comparisons of the sample to a range of population benchmarks and found results consistent with the representativeness of the sample. Details of the KN panel and data generalizability are reported in a technical appendix (17), and the questionnaire is available upon request.

### Variables and analysis

We first assessed how frequently subjects had used the Internet for health information in the last year (more than once a week, about once a week, about once a month, every two to three months, less than every two to three months, never in the last year). We asked about the frequency with which they had searched during the past year, and whether they had used the Internet to communicate about their illness with doctors, other patients, and family or friends. For subjects who said they had used the Internet to find health information, participants were asked to respond "agree," "disagree," or "don't know" to three statements: "it takes too long to find information on the Internet," "I cannot trust information I find on the Internet," and "I can easily understand the information I find on the Internet." We excluded the few (2–6%) who answered "don't know."

We asked questions that referred specifically to one of the respondent's chronic illnesses. The questions asked whether using the Internet or e-mail 1) improved understanding of the illness; 2) improved understanding of possible treatments for the illness; 3) affected the treatments used for the illness; 4) improved the ability of respondent to manage the disease on his or her own; 5) led the respondent to seek care from different doctors or health care providers than respondent otherwise would have; or 6) affected the way respondent ate or exercised. None of the questions asked respondents to recall what information they were seeking. Response categories were "strongly agree," "agree," "disagree," and "strongly disagree"; we collapsed these four into "agree" and "disagree." These six questions were asked only if people reported searching the Internet for health information.

The independent variable of interest was the subject's chronic condition. We asked respondents if a doctor had ever told them that they had 1) high blood pressure or hypertension; 2) diabetes or high blood sugar; 3) cancer; or 4) heart problems, such as a heart attack, coronary heart disease, angina, or heart failure. We also asked whether they had ever had, or had a doctor or other health care provider tell them that they had, depression. For diabetes, respondents could answer "yes," "no," or "borderline" (we recoded borderline as "yes"); for other items, they could answer "yes" or "no."

We used two analytical approaches for comparing the chronic conditions. First, for the dependent variables that did not refer to a specific condition, we created a classification system for the chronic conditions. People could report more than one chronic condition. For most analyses, we classified respondents into one of seven mutually exclusive study groups: hypertension only (n = 505), diabetes only (n = 147), cancer only (n = 59), heart problems only (n = 73), depression only (n = 552), two chronic conditions (n = 451), and three or more (n = 190). We tried developing categories representing different combinations of conditions (e.g., diabetes and hypertension), but making more combinations was intractable and preliminary analysis indicated that doing so would provide little additional information. We note when significant differences between other chronic conditions exist.

We used another approach for questions on the effects of Internet use on the respondent's chronic condition. For people who had more than one chronic condition, we chose one of the conditions randomly and asked all questions about only that one. Again, the chronic condition was the primary variable of interest. But because the questions specifically referred to a chronic condition, we compared hypertension, diabetes, cancer, heart problems, and depression.

We oversampled veterans and older adults (aged more than 50). KN calculated post-stratification sampling weights to reduce the bias due to nonresponse and to reduce sampling variance for characteristics highly correlated with demographic and geographic totals. KN calculated these weights so that the weighted sample cells matched those of the December 2001 U.S. Census Bureau's Current Population Survey. The weights were based on age, veteran status, sex, race/ethnicity, geographic region, metropolitan status, and education.

We weighted all bivariate and multivariate analyses to account for our oversamples of older adults and veterans. The five chronic conditions differed by demographic characteristics that were also associated with using the Internet. Therefore, we used multivariate logistic regression models in which we controlled for education (high school or less, some college, or some graduate school), sex, and age (under 50, 50–64, 65–74, or 75+ years). We treated all control variables as sets of dummy variables to allow for nonlinearities. When testing for statistical significance, we corrected the standard errors for the complex design effects. We conducted all analyses in Stata 8 (StataCorp LP, College Station, Tex).

Although the odds ratios are informative, we were interested in the absolute and relative differences across the chronic conditions. Therefore, we used the logistic regressions to compute predicted probabilities, which we then multiplied by 100 to reflect percentages, using the characteristics of a respondent who had average values of the control variables. In the tables, we present the predicted probabilities based on our multivariate models in which we hold age, education, and sex constant. (The full regression results are presented in Supplemental Tables.)

## Results

Table 1 shows the summary statistics for the sample. People who reported depression only were the largest group (27.9% of the sample). People who had hypertension only constituted the second-largest group (25.5%); people who had two chronic conditions made up the third-largest group (22.8%). The smallest group was people who had cancer only (3.0%).

Of the 641 respondents with more than one chronic condition, 201 (31.4%) reported having hypertension, 171 (26.7%) reported having depression, 116 (18.1%) reported having diabetes, 88 (13.7%) reported having heart problems, and 66 (10.3%) reported having cancer (data not shown). Combinations of two and three chronic conditions comprised 93.0% of this group; 39 (6.1%) people reported four chronic conditions, and only six (1.0%) reported having all five conditions.

### Frequency of Internet use

Among all individuals who had one or more of the five chronic conditions, 45.9% reported using the Internet to seek health information or advice in the past year (Table 2). On average, 11.0% reported at least monthly use. Internet use varied by chronic condition. Those who had hypertension only, cancer only, or heart problems only reported relatively low use (33.8%–42.9%); those who had diabetes only, depression only, or two or more chronic conditions were more likely to use the Internet (47.6%–52.0%).

People with depression only, cancer only, and three or more chronic conditions were more likely to use e-mail or other Internet-based services to communicate with heath professionals than people with hypertension only or heart problems only (*P *<.05). Using the Internet to communicate with family or friends was the most common form of communication, ranging from approximately 26.6%–41.7%, except for people with diabetes only (16.6%). People with three or more chronic conditions or cancer only reported higher rates of communicating with other patients, compared with people with hypertension only or heart problems only (*P *<.05).

### Attitudes about the Internet

Among people who used the Internet for health information, 38.7% agreed that it takes too long to find information on the Internet, 20.7% agreed that they cannot trust information on the Internet, and 82.6% agreed that the information on the Internet is easy to understand. There were no statistically significant differences across study groups, except for the finding that 18.7% of people in the heart-problems–only group agreed that it takes too long to find information, compared with diabetes only (48.7%, *P* < .01), depression only (42.4%, *P* <.01), two chronic conditions (40.6%, *P* <.05), and three or more chronic conditions (42.1%, *P* <.05).

### Self-reported effects

When stratified by condition, nearly one half to more than three quarters of respondents reported that Internet use or e-mail improved their understanding of their condition(s) (Table 3). Approximately the same percentage of respondents said that Internet use improved their understanding of possible treatments. People who had diabetes and heart problems responded positively more frequently than did those who had hypertension (*P* < .05).

A much smaller percentage in all groups reported that the Internet affected the treatment(s) they received for their condition(s). Although 23.5% of people with diabetes and 26.9% of people with heart problems said Internet use affected their treatments, only the latter value was marginally statistically greater than for people who had hypertension (15%, *P* = .06).

Overall, 28.3% of respondents with one of five chronic conditions reported that Internet information had improved their ability to manage their condition by themselves. People who had diabetes reported most frequently that the Internet had improved their ability to manage their condition (38.4%), and this was statistically greater than for people with depression (22.3%, *P* < .01), but was not greater than for the other chronic condition groups. Fewer than one in eight reported that the Internet had led them to seek care from different doctors or providers; there were no significant differences across the conditions. When we asked about whether the Internet had affected the way that subjects ate or exercised, 49.2% of those with diabetes said yes, a significantly greater proportion than the proportion of people who had depression (31.0%, *P* < .01) or cancer (29.7%, *P* < .05).

## Discussion

People with chronic conditions vary in their use of the Internet for health information and advice. After adjusting for education, sex, and age, Internet use for health information in the past year clustered at about 33.8% to 42.9% for hypertension only, cancer only, and heart problems only. Rates were slightly higher (47.6%) for people with any two of the five chronic conditions and were 51.0% to 52.0% for people with diabetes only, depression only, and three or more chronic conditions.

People who have depression only and people who have multiple chronic conditions were among the most frequent users of the Internet for health information — for overall use and for communicating with health professionals. Use of the Internet was also common for people with diabetes only, although these people were less likely to communicate with family and no more or less likely to communicate with health professionals or other patients than the other chronic condition groups. These data confirm past reports that people who have depression are more likely to seek health information than people who have other chronic conditions (13,18). The higher rates for depression only may reflect that the disorder still carries some stigma, leading individuals to seek information outside traditional routes. The higher rates for depression could also reflect that depression care often has greater limits on mental health care insurance benefits and higher out-of-pocket expenses. It would be useful to know whether this association is driven by stigma or by costs. If the association is driven by stigma, this could identify opportunities for using the Internet to reach people with stigmatized and potentially communicable chronic conditions (e.g., HIV).

Although a large percentage of people with depression searched the Internet only for health information, the depression-only group reported one of the lowest rates for having been affected by Internet use. People who had diabetes, cancer, or heart problems were more likely to agree that Internet information improved their understanding of their condition than were people who had hypertension or depression. More research is needed to determine which type of information is received by people who have depression and whether they find it helpful.

We interpret Internet use among people with chronic conditions as a glass half empty or half full. We see smaller effects on treatments and providers and larger effects on self-management, eating, and exercise. Fewer than one in eight people agreed that the Internet led them to seek care from different health professionals for their conditions, and fewer than one in four said that the Internet affected their choice of treatment. These numbers can be viewed as substantial or meager, depending upon perspective. If these numbers are accurate, the effect of the Internet on improved understanding is larger than other computerized patient education interventions, such as the one described in the study by Rostom et al on decision support for hormone replacement therapy (19) or in the study by Consoli et al on hypertension (20). Caution must be used in interpreting these responses because the data are self-reported, and we do not have information about respondents' knowledge before they used the Internet and cannot compare these data to a control group. We also have no way of verifying if the information they obtained was factually correct.

Attitudes toward health information on the Internet were generally favorable. Slightly more than one third of the people with one or more of the five chronic conditions agreed that it takes too long to find information on the Internet, indicating that search time is an important determinant in using the Internet. When people do find information, they then have to identify whether it is high quality and accurate. Approximately one in five people agreed that they cannot trust Internet health information, but it is unclear how they determine whether they can trust the information. Other studies have discussed problems with the quality and coverage of health information on the Internet (6,8,21), and research has found that people are not particularly good judges for identifying accurate health information and often forget which sites they searched (7). Efforts to help people identify high-quality information more quickly could result in more people using the Internet for health information.

A limitation of this study was that all the data were self-reported. Some respondents might have avoided labeling themselves as chronically ill, especially for depression, which is stigmatized. Additionally, the survey questions required that people reconstruct memories of how they used the Internet in the past year. This process can be cognitively difficult, especially when a question asks respondents to remember how they used the Internet and then to estimate its net effect.

The KN panel has been used in other research studies (22,23). This method of sampling departs from traditional random-digit dialing. Both methods start with a sampling frame that consists of U.S. households with telephone access. Both methods have strengths and weaknesses. The strength of the panel approach is that people are asked to participate in the panel, and a subset is sampled for a particular survey. We have information on those who were sent the survey and did not respond. The weakness is that some people may dislike being on a panel and opt out when first asked or ask to be removed from the panel over time. KN and independent researchers have studied these issues and reports are available online (http://www.knowledgenetworks.com/ganp/reviewer-info.html). Random-digit dialing is performed each time a survey is fielded, so it is not susceptible to panel attrition. At the onset of the call, however, the respondent is told about the intent of the survey. People then choose whether to complete the survey, and, typically, little if any information on the non-respondents is collected. Many national surveys, including the Behavioral Risk Factor Surveillance Survey and 2000 Census, report median response rates below 70%. The latest study on the Internet and health conducted by Pew Internet and American Life Project reported a response rate of 32.8% (15). As mentioned earlier, we compared our sample to other national surveys. Although the results were similar on all the variables we compared (17), we cannot rule out the existence of potential biases on other variables, such as Internet use.

This study focuses on common chronic conditions. Perhaps we would see higher rates of Internet use among people who have rare diseases. There is a substantial amount of health information available on the Internet (24), and people with rare illnesses can obtain peer support on the Internet in ways that would not be possible off-line. Further research could evaluate these matters.

A common perception is that the provision of health information via the Internet is a "field of dreams" — that is, if we build it, they will come. In the past decade, public and private investments have poured into Internet sites. Although the Internet can offer several clear advantages over traditional information sources, such as very low distribution costs, we found that few people who have the five common chronic conditions studied use it routinely. When they do, however, they report notable gains in knowledge and small changes in behavior.

## Figures and Tables

**Table 1:** Characteristics of Participants, Survey on Using the Internet for Health Information (n = 1980), United States, 2001–2002^a^

	**n**	**%**	**SE (%)**

**Chronic Condition**			

Hypertension only	505	25.5	1.4
Diabetes only	147	7.4	0.9
Cancer only	59	3.0	0.4
Heart problems only	73	3.7	0.6
Depression only	552	27.9	1.6
Two chronic conditions	451	22.8	1.4
Three or more chronic conditions	190	9.6	0.8

**Age (years)**			

<50	1131	57.1	1.5
50-64	586	29.6	1.3
65-74	196	9.9	0.7
75+	65	3.3	0.4

**Education**			

<13 years	834	42.1	1.6
13-16 years	941	47.5	1.6
17+ years	208	10.5	0.8
**Female**	1154	58.3	1.6

**Total**	1980		


a All data are weighted.

**Table 2:** Use of Internet for Health Information, Survey Results (n = 1980), United States, 2001–2002^a^

	**Used Internet for Information or Advice About Health or Health Care**		**Used E-mail or Internet in Past Year to Communicate With**		

	**Ever in last year**	**At least monthly**	**Health professional**	**Family and friends**	**Other patients**

**Chronic Condition**					

Hypertension only	39.6	8.5	4.1	26.6^d^	10.9
Diabetes only	52.0	14.7	5.6	16.6	13.3
Cancer only	42.9	16.6^c^	11.0^b^,^c^	29.8^d^	16.8^c^
Heart problems only	33.8	9.5	3.7	29.3^d^	5.6
Depression only	51.0	8.8^c^	10.5^b^,^c^	31.7^d^	13.5
Two chronic conditions	47.6	12.7^c^	6.6	30.0^d^	12.3
Three or more chronic conditions	51.5^b^,^c^	15.3^b^,^c^	9.3^b^,^c^	41.7^b^,^d^	25.6^b^,^c^

**Overall**	45.9	11.0	6.6	30.0	13.1


a All figures are percentages. Percentages were calculated from a logistic regression model that included age, sex, and education, and that controlled for the complex design effects. Some totals may not sum to 100% because of rounding.

b Significant at 5% (two-tailed) compared to hypertension only.

c Significant at 5% (two-tailed) compared to heart problems only.

d Significant at 5% (two-tailed) compared to diabetes only.

**Table 3:** Self-reported Effects of Using the Internet for Health Information, Survey Results, United States, 2001–2002^a^

	**Improved my under-** **standing of X** **(n=851)**	**Improved my under-** **standing of possible treatments for X** **(n=851)**	**Affected the treat-** **ments I am using for X** **(n=843)**	**Improved my ability to manage my X by myself** **(n=831)**	**Led me to seek care from different doctors or health care providers for X than I otherwise would have** **(n=837)**	**Affected the way I eat or exercise** ** (n=847)**

**Chronic Condition**						

Hypertension	48.1	45.3	15.0	29.1	6.2	39.6
Diabetes	66.6^b^	67.0^b^	23.5	38.4	8.0	49.2
Cancer	62.9	59.3	14.5	25.6	12.2	29.7^c^
Heart problems	79.3^b^	75.8^b^	26.9	32.6	11.6	35.0
Depression	47.1	45.4	17.8	22.3^c^	8.3	31.0^c^

**Overall**	55.8	53.4	17.9	28.3	7.9	36.9


a All figures are percentages. Percentages were calculated from a logistic regression model that included age, sex, and education, and controlled for the complex design effects. People with more than one of the five chronic conditions were randomly assigned to one condition. X indicates a chronic condition.

b Significant at 5% (two-tailed) compared to hypertension only.

c Significant at 5% (two-tailed) compared to diabetes only.

## Appendix. Validity of the Survey of Health and Internet and Knowledge Network's Panel and Sampling

Our study was conducted using an Internet-based survey methodology that may be unfamiliar to reviewers. The appendix addresses potential questions reviewers may have. In particular, we provide more detailed information about Knowledge Networks (KN), the survey research firm conducting the survey, the techniques used in the survey, and results of analyses performed by KN and other researchers, including the authors, regarding the validity of the methodology.

[Note from the editor: For our readers' convenience, we have converted this appendix into downloadable PDF format. The PDF format simplifies the printing process and maintains the appearance of the original document.]

Validity of the Survey of Health and Internet and Knowledge Network's Panel and Sampling (PDF 567K)

## Supplemental Tables

**Supplemental Table A:** Internet Use for Health Information in Last Year, Regression Results, United States, 2001–2002^a^

	**Used Internet in Last Year for Health Information** **OR (95% CI)**	**Used Internet Monthly for Health Information** **OR (95% CI)**	**Communicated With Doctor in Last Year on Health Information** **OR (95% CI)**	**Communicated With Family Last Year on Health Information** **OR (95% CI)**	**Communicated With Other Patients in Last Year on Health Information** **OR (95% CI)**

**Chronic Condition**					

**Hypertension only**	Ref	Ref	Ref	Ref	Ref
**Diabetes only**	1.564 (0.882-2.773)	1.713 (0.716-4.097)	1.429 (0.392-5.205)	0.518^b^(0.277-0.972)	1.208 (0.496-2.945)
**Cancer only**	1.174 (0.649-2.124)	2.338 (0.946-5.781)	2.990^b^ (1.045-8.556)	1.244 (0.618-2.501)	1.763 (0.695-4.475)
**Heart problems only**	0.749 (0.375-1.496)	1.172 (0.442-3.105)	0.842 (0.342-2.070)	1.162(0.523-2.577)	0.514 (0.192-1.374)
**Depression only**	1.439 (0.987-2.099)	0.83 (0.451-1.527)	2.971^c^ (1.427-6.187)	1.092 (0.722-1.652)	1.122 (0.626-2.011)
**2 chronic conditions**	1.409 (0.970-2.048)	1.648 (0.899-3.018)	1.73 (0.778-3.846)	1.198 (0.802-1.789)	1.169 (0.663-2.060)
**3+ chronic conditions**	1.658^ b^ (1.050-2.616)	2.091^b^ (1.039-4.208)	2.518^ b^ (1.191-5.326)	2.025^c^ (1.256-3.267)	2.923^c^ (1.549-5.514)

**Age (years)**					

**<50 **	Ref	Ref	Ref	Ref	Ref
**50-64**	1.142 (0.857-1.520)	0.729 (0.474-1.121)	0.994 (0.566-1.745)	1.091 (0.801-1.485)	0.904 (0.595-1.374)
**65-74**	1.023 (0.689-1.518)	0.656 (0.375-1.147)	0.787 (0.379-1.636)	0.763 (0.505-1.153)	0.626 (0.341-1.150)
**75+**	0.544^ b^ (0.323-.916)	0.533 (0.231-1.230)	0.946 (0.388-2.304)	0.654 (0.370-1.157)	0.765 (0.375-1.562)

**Education (years)**					

**<13**	0.368^ c^ (0.245-0.552)	0.599 (0.330-1.087)	0.638 (0.329-1.234)	0.624^ b^ (0.409-0.950)	0.968 (0.578-1.624)
**13-16 **	0.813 (0.562-1.176)	0.987 (0.587-1.662)	0.914 (0.529-1.580)	0.984 (0.674-1.436)	0.878 (0.547-1.411)
**>16**	Ref	Ref	Ref	Ref	Ref

**Sex**					

**Female**	1.679^ c^ (1.297-2.173)	1.599^ b^ (1.054-2.427)	0.705 (0.418-1.190)	1.659^ c^ (1.248-2.206)	1.203 (0.813-1.779)

**Observations**					

**Weighted**	1980	1980	1980	1980	1980
**Unweighted**	2391	2391	2391	2391	2391

a OR indicates odds ratio; CI indicates confidence interval; Ref indicates reference group. Regressions controlled for the complex design effects.

b Significant at 5%.

c Significant at 1% (two-tailed).

**Supplemental Table B:** Perceptions of Using the Internet Among Individuals Who Use the Internet for Health Information, Regression Results, United States, 2001–2002^a^

	**Agree that "It takes too long to find information on Internet"** **OR (95% CI)**	**Agree that "I cannot trust health information on Internet"** ** OR (95% CI)**	**Agree that "I can easily understand health information on Internet" ** **OR (95% CI)**

**Chronic Condition**			

**Hypertension only**	Ref	Ref	Ref
**Diabetes only**	1.826 (0.804-4.145)	2.133 (0.822-5.537)	1.175 (0.397-3.480)
**Cancer only**	1.094 (0.441-2.715)	0.909 (0.337-2.449)	1.664 (0.624-4.435)
**Heart problems only**	0.42 (0.171-1.032)	0.649 (0.201-2.097)	1.037 (0.249-4.328)
**Depression only**	1.532 (0.873-2.689)	1.802 (0.880-3.689)	1.116 (0.570-2.186)
**2 chronic conditions**	1.217 (0.703-2.106)	1.566 (0.762-3.219)	1.325 (0.701-2.503)
**3+ chronic conditions**	1.26 (0.677-2.342)	1.259 (0.586-2.702)	0.959 (0.490-1.878)

**Age (years)**			

**<50**	Ref	Ref	Ref
**50-64**	1.14 (0.756-1.719)	0.639 (0.382-1.069)	0.825 (0.505-1.348)
**65-74**	1.619 (0.914-2.869)	0.495^b^ (0.249-0.984)	1.315 (0.656-2.634)
**75+ **	1.819 (0.841-3.938)	0.652 (0.269-1.580)	1.558 (0.604-4.017)

**Education (years)**			

**<13**	1.414 (0.807-2.477)	1.37 (0.706-2.659)	0.897 (0.460-1.747)
**13-16**	1.214 (0.758-1.946)	1.348 (0.782-2.323)	1.232 (0.709-2.140)
**>16**	Ref	Ref	Ref

**Sex**			

**Female**	0.927 (0.625-1.374)	0.557^b^ (0.344-0.901)	1.294 (0.817-2.049)

**Observations**			

**Weighted**	894	887	893
**Unweighted**	1125	1119	1120

a OR indicates odds ratio; CI indicates confidence interval; Ref indicates reference group. Regressions controlled for the complex design effects.

b Significant at 5%.

**Supplemental Table C:** Effects of Using Internet for Health Information, Regression Results, United States, 2001^a^

	**"Improved my understanding of X"** ** OR (95% CI)**	**"Improved my understanding of possible treatments for X"** **OR (95% CI)**	**"Affected the treatments I am using for X"** **OR (95% CI)**	**"Improved my ability to manage my X by myself"** **OR (95% CI)**	**"Led me to seek care from different doctors or health care providers for X than I otherwise would have"** **OR (95% CI)**	**"Affected the way I eat or exercise"** **OR (95% CI)**

**Chronic Condition**						

**Hypertension**	Ref	Ref	Ref	Ref	Ref	Ref
**Diabetes only**	2.137^b^ (1.146-3.986)	2.397^c^ (1.291-4.452)	1.825 (0.842-3.953)	1.534 (0.787-2.990)	1.427 (0.442-4.602)	1.462 (0.781-2.735)
**Cancer only**	1.801 (0.865-3.748)	1.723 (0.828-3.585)	0.936 (0.406-2.157)	0.942 (0.421-2.105)	2.187 (0.788-6.072)	0.675 (0.333-1.367)
**Heart problems only**	4.174^c^ (2.275-7.658)	3.795^c^ (2.052-7.019)	2.046 (0.964-4.342)	1.27 (0.595-2.712)	2.044 (0.762-5.483)	0.831 (0.435-1.587)
**Depression only**	0.945 (0.590-1.512)	0.969 (0.604-1.554)	1.343 (0.750-2.404)	0.638 (0.364-1.118)	1.432 (0.588-3.488)	0.685 (0.420-1.119)

**Age (years)**						

**<50 **	Ref	Ref	Ref	Ref	Ref	Ref
**50-64**	0.803 (0.537- 1.201)	0.762 (0.511- 1.136)	1.274 (0.778-2.085)	0.797 (0.514-1.237)	1.044 (0.515-2.117)	0.93 (0.618-1.399)
**65-74**	1.102 (0.615- 1.973)	1.045 (0.582- 1.877)	1.184 (0.582-2.407)	0.617 (0.319-1.195)	0.641 (0.252-1.633)	1.324 (0.732-2.392)
**75+**	0.916 (0.393- 2.131)	0.992 (0.423- 2.323)	1.487 (0.513-4.307)	0.64 (0.232-1.763)	1.739 (0.445-6.795)	0.402^b^ (0.181-0.894)

**Education (years)**						

**<13**	1.094 (0.609-1.967)	1.003 (0.558- 1.806)	1.385 (0.688- 2.788)	1.762 (0.923-3.365)	1.723 (0.649-4.577)	1.09 (0.607-1.960)
**13-16**	0.993 (0.596-1.656)	1.105 (0.663-1.842)	1.011 (0.555- 1.839)	1.238 (0.716- 2.140)	0.942 (0.411-2.162)	1.115 (0.689-1.804)
**>16**	Ref	Ref	Ref	Ref	Ref	Ref

**Sex**						

**Female**	1.014 (0.684-1.504)	1.08 (0.728-1.604)	0.856 (0.504- 1.453)	0.866 (0.551- 1.361)	0.703 (0.321-1.540)	0.897 (0.596-1.350)

**Observations**						

**Weighted**	851	851	843	831	837	847
**Unweighted**	1069	1069	1055	1050	1053	1059

a OR indicates odds ratio; CI indicates confidence interval; Ref indicates reference group; X indicates survey participant’s chronic condition. Regressions controlled for the complex design effects.

b Significant at 5%.

c Significant at 1% (two-tailed).
