# Supplementary material for: Use of the Internet for Health Information by the Chronically Ill
Source: Prev Chronic Dis. 2004 Sep 15;1(4):A13. (PMC1277953)
Supplement: Supplementary file 1 — Validity of the Survey of Health and Internet and Knowledge Network's Panel and Sampling (PDF 567K) [file 04_0004_01.pdf]

Validity of the Survey of Health and Internet and Knowledge Network's Panel and Sampling

Survey of Health and the Internet

Funded by National Institute on Aging
Department of Veterans Affairs &
Stanford University

May 2003

1. Introduction

Our study was conducted using an internet-based survey methodology that may be unfamiliar to reviewers. In this document, we address potential questions reviewers may have. In particular, we provide more detailed information about Knowledge Networks (KN), the survey research firm conducting the survey, the techniques used in our survey and results of analyses performed by KN and other researchers, including ourselves, regarding the validity of the methodology.

2. Previous Publications Using the KN Panel

Perhaps the strongest generalized endorsement of surveys conducted using the KN panel is that the panel is increasingly used by top researchers in a range of fields, including health care, publishing in top journals. For example, in the health field, data based on KN surveys, using essentially the same methodologies we use in our study, have been used in publications in JAMA,[1] the Morbidity & Mortality Weekly Report,[2] Health Services Research,[3] Personality and Social Psychology Bulletin,[4] and the Journal of Personality and Social Psychology.[5]

3. Overview of Knowledge Networks and Survey Administration

We administered our survey to a sample drawn from the KN panel. KN is a survey firm that maintains a large panel of internet-enabled survey respondents. The KN panel is recruited through random digit dialing (RDD). Specifically, the panel sample is selected using a list-assisted RDD telephone methodology, providing a probability-based starting sample of U.S. telephone households. The sample frame is updated quarterly. Knowledge Networks excludes only those banks of telephone numbers (consisting of 100 telephone numbers) that have zero directory-listed phone numbers. Knowledge Networks' telephone numbers are selected from the 1+ banks with equal probability of selection for each number. Note that the sampling is done without replacement to ensure that numbers already fielded by Knowledge Networks do not get fielded again. Having generated the initial list of telephone numbers, the sample preparation system excludes confirmed disconnected and non-residential telephone numbers.

These households are contacted and invited to join the panel. Cases sent to telephone interviewers are dialed up to 90 days, with at least 15 dial attempts on cases where no one answers the phone, and 25 dial attempts on phone numbers known to be associated with households. Extensive refusal conversion is also performed.

KN provides households in the panel with free Web access and an internet appliance (MSN WebTV), which uses a telephone line to connect to the internet and uses the television as a monitor. In return, panel members participate in 10- to 15-minute internet surveys three to four times a month.

To ensure consistent delivery or survey content, each household is provided with identical hardware, even if they currently own a computer or have Internet access. Microsoft's WebTV is the hardware platform currently used by the Knowledge Networks panel. (Please note that currently Knowledge Networks is experimenting with allowing some panel members to use their own personal computers for survey taking, but this innovation occurred after this study was conducted.) The device consists of a set-top box that connects to a TV and the telephone. It also includes a remote keyboard and pointing device. WebTV has a built-in 56K modem that provides the household with a connection to the Internet. The base unit also has a small hard drive to accommodate large file downloads, including video files. File downloads do not require any user intervention and usually occur during off hours.

---

[1] Schlenger, W. E., J. M. Caddell, et al. (2002). "Psychological reactions to terrorist attacks: Findings from the national study of Americans' reactions to September 11." JAMA 288(5): 581-588; Silver, R. C., E. A. Holman, et al. (2002). "Nationwide longitudinal study of psychological responses to September 11." JAMA 288(10): 1235-1244.
[2] Lentine, D. A., J. C. Hersey, et al. (2000). "HIV-related knowledge and stigma - United States, 2000." Morbidity and Mortality Weekly Report 49(47): 1062-1064.
[3] Harris, K. M. (forthcoming). "How do patients choose physicians? Evidence from a national survey of enrollees in employment-related health plans." Health Services Research.
[4] Skitka, L. J. and E. Mullen (2002). "Understanding judgments of fairness in a real-world political context: a test of the value protection model of justice reasoning." Personality and Social Psychology Bulletin. 28(10): 1419-1429
[5] Skitka, L. J., E. Mullen, et al. (2002). "Dispositions, scripts, or motivated correction? Understanding ideological differences in explanations for social problems." Journal of Personality and Social Psychology 83(2): 470-487.

Prior to shipment, each unit is custom configured with individual email accounts, so that it is ready for immediate use by the household. Most households are able to install the hardware without additional assistance, though Knowledge Networks maintains a telephone technical support line and will, when needed, provide on-site installation. The Knowledge Networks Call Center also contacts household members who do not respond to e-mail and attempts to restore contact and cooperation.

All new panel members are sent an initial survey to confirm equipment installation and familiarize them with the WebTV unit. After the initial survey, surveys are conducted by sampling from the panel and sending a questionnaire to respondents via the provided internet access point. Survey responses are confidential, with identifying information never revealed without respondent approval. When surveys are assigned to panel members, they receive notice in their password-protected email account that the survey is available for completion. Surveys are self-administered and accessible any time of day for a designated period. Participants can complete a survey only once. Members may leave the panel at any time, and receipt of the internet service is not contingent on completion of any particular survey.

While some internet-based survey methodologies involve sampling only from the set of individuals who made a decision to obtain internet access on their own, the KN methodology does not. Moreover, many internet-based surveys recruit their sample on line, with likely resulting in a sample that includes a disproportionate number of enthusiastic internet users who are the most likely to be on line to be recruited. The KN methodology, which surveys individuals randomly selected to receive internet access, is not affected by this type of bias. In the end, while the KN methodology uses the internet for survey administration, the panel and the sampling methodology is very different than all other internet-based surveys.

Evidence suggests that the KN panel is generally free from the most common types of biases found in other internet methodologies. The entire web-enabled panel tracks closely the U.S. population on age, race, Hispanic ethnicity, geographical region, employment status, and other demographic elements. Table A1 presents information comparing the entire KN panel and active participants in the KN panel to the U.S. population using Census data using June 2002 data.[6] The differences that do exist are small.

4. Studies of Aspects of KN Panel Validity

A number of previous analyses support the validity of administering surveys using the KN panel. One issue is whether non-response patterns among those who are approached to join the panel produces a non-representative group of panel members. Existing work suggests that this is not a significant concern. In one recent study, the "Survey on Health and Aging"[7] researchers surveyed panel members via internet and also surveyed by telephone a group of individuals in households who did not respond to the panel recruitment efforts. They found evidence consistent with the view that responses from just the panel members were statistically strong. Adding in data from the telephone survey of those who did not respond to panel recruitment efforts did not affect the extent to which the sample was representative of the population and did not substantively influence the results of the analysis.

A related matter is the extent to which there is attrition from the panel. If the original panel is statistically valid, but there is disproportionate attrition from the panel among some groups, the panel could become biased over time. Analyses of panel attrition patterns suggests that substantive survey results are minimally affected by panel attrition. For example, KN looked at responses to 30 survey questions from health and political profile surveys administered to panel members shortly after they joined the panel. They compared responses among the groups of individuals who remained on the panel over relatively long periods and groups who dropped off the panel. Large differences in these estimates would indicate that exit patterns among panel members could cause the overall panel to become unrepresentative over time. However, the average absolute difference observed across the variables examined was small (0.009, or less than percentage point), consistent with the view that panel attrition was not disproportionately centered in groups with certain characteristics and, at least in this case, had a negligible impact on data quality. We note that many of the health questions that were part of this analysis are similar to the kinds of questions and issues we examine in our analyses.

[6] These data from personal communication with J.M. Dennis of Knowledge Networks, September 9, 2002.
[7] See Wiebe, E.F., J. Eyerman, and J. Loft, *Evaluating nonresponse in a Web-enabled survey on health and aging*. 2001, Presented at the 2001 Meeting of the American Association for Public Opinion Research, Montreal, Quebec, May 17-20. Available from: http://www.knowledgenetworks.com/ganp/safe/aapor%202001%20nonresponse.pdf. Accessed September 30, 2002.

Finally, work has compared the use of the KN panel to telephone surveys using RDD, as performed by many survey organizations. Work by Krosnick and Chang[8] commissioned side-by-side surveys of public opinion and voting intentions for the 2000 U.S. Presidential election using the KN panel and using a RDD household sample (among other survey modes). They found that the KN panel and the RDD survey were the best performers of the modes they examined in terms of generating responses from populations that matched the U.S. census measures of demographics and producing consistent measures on the substantive survey questions that correlate with political attitudes and behaviors obtained from other studies.

5. Panel Acceptance, Attrition and Survey Completion Rates

Readers and researchers may be accustomed to interpreting response rates from random digit dial (RDD) surveys. However, there are important distinctions when using panels for survey research. It is important to understand that panels have a dynamic nature and potential bias can enter at more than one point in time. First, people agree to become panel members. We refer to this as the panel acceptance rate. At the time of our survey, the panel acceptance rate was 41.2%, calculated by standards established by the American Association for Public Opinion Research.[9] Second, attrition from the panel happens over time. At the time of our survey, of the recruited sample, 35.5% of the recruited panel was active and available for selection at the time of selecting the sample for this study.[10] Finally, there is the survey completion rate—or percentage of panel members who completed the questionnaire among all of those who were sent the questionnaire. In total, KN administered the survey to a sample of 12,878 panel members. Of the 12,878 sent the questionnaire, 2,265 (18%) persons declined consent, 1,678 persons (13%) did not complete the consent form, and 8,935 (69%) provided informed consent and subsequently completed the survey.

6. Weights

After the survey was completed, appropriate sample design weights were calculated based on specific design parameters. Finally, nonresponse and poststratification weighting adjustments are applied to the final survey data to reduce the effects of nonsampling error (variance and bias). The following variables for poststratification weighting:

- age: 21-29, 30-44, 45-49, 50 and over
- Veteran status
- gender: male, female
- race/ethnicity: white (nonhispanic), black (nonhispanic), other (nonhispanic), hispanic
- region: northeast, midwest, south, west
- metropolitan status: metropolitan, nonmetropolitan
- education - highest level achieved: less than high school, high school, some college, college degree or more

To calculate final weights, KN derived weighted sample distributions along various combinations of the above variables. Similar distributions were calculated using the most recent U.S. Census Bureau's Current Population Survey (December 2001). Cell-by-cell adjustments over the various univariate and bivariate distributions were calculated to make the weighted sample cells match those of the U.S. Census and the Knowledge Networks Panel. This process, known as raking, is repeated iteratively until there is convergence between the weighted sample and benchmark CPS distributions. Occasionally, collapsing of post-stratification cells is necessary. This is dependent on the size of the sample and topology of the sample universe. The final weights were trimmed at ± 2.5% and scaled to the complete and qualified sample size.

KN also provided a variable in the data file at the panel member level that reflects the sample design for the Knowledge Networks Panel. This variable can be used to calculate corrected variance estimates in Sudaan or Stata.

---

[8] Krosnick, J. A. and L. Chang (2001). A comparison of random digit dialing telephone survey methodology with internet survey methodology as implemented by Knowledge Networks and Harris Interactive. Ohio State University.

[9] American Association for Public Opinion Research. Standard definitions: final dispositions of cases and codes and outcome rates for surveys. Available at http://www.aapor.org/ethics/stddef.html.

[10] KN has done research on attrition, see http://www.knowledgenetworks.com/ganp/aapor2003.pdf.

7 Analysis of the Validity of the Survey of Health and the Internet

Samples for any particular survey are drawn from the panel. Care is taken in sampling for any particular survey to ensure that samples drawn from the overall panel are representative, using information from U.S. Census demographic benchmarks. Among other things, this helps to reduce error due to noncoverage of nontelephone households and to reduce bias due to nonresponse and other nonsampling errors.

Table A2a shows how our *unweighted* survey data compare to CPS with regard to gender, age, marital status, household race/ethnicity, education, household income and region. Table A2b shows how our *weighted* survey data compare to the CPS on the same variables. In most cases the differences are quite small, and the average is approximately 2-3%. The KN sample has fewer people with a household income of over $75,000 and more people with an income between $25,000 and $50,000.

Our particular survey focuses on health care issues. Another way of establishing the representativeness of the panel for health work is to compare health care measures for panel members to measures obtained from other sources, such as the widely respected National Health Interview Survey (NHIS). The NHIS is conducted in-person using a high-quality area probability sample of telephone and nontelephone households. Table A3 presents results from an analysis comparing panel characteristics as measured by a baseline Knowledge Networks survey (not our survey) to U.S. population characteristics from the 2000 NHIS, in cases where similar questions were available for both groups.[6] As shown in Table A3, the results are similar on the measures compared: past smoking ("ever"), current smoking, diabetes, ulcer, migraine headaches, and stroke. As above, differences are small.

We performed several analyses with the group of responders to our survey to investigate the extent to which observable characteristics of our sample are comparable to characteristics of the U.S. population as measured on other highly regarded national surveys.

First, we examined the self-reported prevalence rates of hypertension, heart problems, cancer, and diabetes, all of which are measured in our survey and in the 2000 NHIS. In the NHIS data, we limit analyses to respondents who were 21 and over for comparison with our survey population. We also only use NHIS respondents who are in the "sample adult" file, the source of the self-reported health condition information. We weighted our results and the NHIS results using weights appropriate for production of nationally representative results. Table A4 reports results. The reported condition prevalences are generally similar for the conditions studied.

Next, we compared rates of use of health care providers using the same sample restrictions and weighting. Results of comparisons between our survey and the 2000 NHIS are shown in Table A5. Rates of health care provider office visits and emergency room visits are generally similar.

As a second comparison for office visits, we examined provider visit data from the 1999 Center For Studying Health Systems Change national Community Tracking Study Household Survey (CTSHS). We used CTSHS data from the augmented site sample, and weighted appropriately for computing national estimates. We used responses from only the "reference person", who provided information about himself or herself, for comparability with our survey where individuals responded for themselves. We included only responses from individuals 21 years of age and over. Results are shown in Table A6. As in the comparison with the NHIS, rates of office visits and ER visits are similar in the two surveys.

We compared measures of the number of hospitalizations in our data to measures from the 2000 NHIS and 1999 CTSHS, using the same sample selection and weighting approaches. Results are in Table A7, again generally similar across the surveys.

In addition to the analyses shown, we also examined a handful of other measures where the survey questions were not as well matched across the surveys, including visits to mental health professionals, visits to chiropractors, and smoking status. Even for these questions, we found that concordance between our survey and the national surveys was high.

When we examined self-reported health status, though, we did find somewhat fewer people reporting their health as "excellent" and somewhat more reporting their health as "good" on our survey compared to the NHIS and CTS benchmarks. In summary, however, we examined many different characteristics of survey respondents and found that in the vast majority of cases, the estimates were consistent with those produced by other surveys.

Table A1: Comparison of KN Panel Characteristics to U.S. Census Data

Characteristics		U.S Census (CPS, Feb. 2002)	All Profiled KN Members (June 2002)	Active KN Members (June 2002)
Gender	Male	48.0%	48.0%	47.9%
	Female	52.0%	52.0%	52.1%
Age	18-29	21.7%	21.6%	21.3%
	30-44	31.1%	31.1%	31.0%
	45-59	25.8%	27.0%	27.0%
	60+	21.4%	20.3%	20.7%
Race/Ethnicity	White	72.7%	72.8%	72.9%
	Black	11.6%	11.5%	11.4%
	Other	4.7%	4.8%	4.8%
	Hispanic	11.0%	10.9%	10.9%
Employment Status	In labor force	64.0%	68.3%	65.2%
	Working full time	53.2%	57.4%	53.5%
	Working part time	10.8%	10.9%	11.7%
	Not in labor force	36.0%	31.7%	34.8%
Marital Status	Married	57.3%	60.5%	61.1%
	Not married	42.7%	39.5%	38.9%
Housing ownership	Own	N/A	69.5%	66.2%
	Rent/Other	N/A	30.5%	33.7%
Household income	Under $10,000	7.5%	6.5%	8.1%
	$10,000 - $24,999	18.5%	15.7%	18.1%
	$25,000 - $49,999	29.2%	35.4%	34.8%
	$50,000 - $ 74,999	19.9%	23.3%	21.2%
	$75,000 or more	24.9%	19.1%	17.8%
Education	Less than HS	16.4%	16.7%	16.7%
	High School	32.0%	32.3%	32.3%
	Some college	27.4%	27.0%	27.0%
	College	24.3%	24.0%	24.0%
Region	Northeast	19.1%	19.3%	19.2%
	Midwest	22.8%	22.7%	22.9%
	South	35.6%	35.4%	35.3%
	West	22.6%	22.6%	22.6%

*CPS data are weighted. KN data are weighted by panel design weights and raking variables employed for survey sampling.

Table A2a: Frequencies of CPS December 2001 compared to our Unweighted KN survey data (fielded December 2001-January 2002)

	CPS December 2001			KN Unweighted Distributions of Completes			Differences KN Unweighted - CPS		
	Age 21+	Age 50+	Veterans 21+	Age 21+	Age 50+	Veterans 21+	Age 21+	Age 50+	Veterans 21+
Gender									
Male	47.8%	45.8%	94.4%	46.82%	45.82%	96.24%	-1.0%	0.0%	1.8%
Female	52.2%	54.2%	5.6%	53.18%	54.18%	3.76%	1.0%	0.0%	-1.8%
Age range									
21-24	7.9%	0.0%	1.1%	4.68%		0.40%	-3.2%	0.0%	-0.7%
25-34	19.1%	0.0%	7.1%	17.31%		6.05%	-1.8%	0.0%	-1.1%
35-44	22.9%	0.0%	12.3%	22.99%		14.78%	0.1%	0.0%	2.5%
45-54	20.1%	23.8%	20.9%	23.21%	20.91%	24.27%	3.1%	-2.9%	3.4%
55-64	12.9%	32.7%	20.8%	15.52%	37.99%	21.11%	2.7%	5.3%	0.3%
>65	17.1%	43.6%	37.8%	16.29%	41.10%	33.40%	-0.8%	-2.5%	-4.4%
Marital status									
Married	60.2%	63.9%	72.4%	64.19%	67.63%	74.94%	4.0%	3.8%	2.6%
Single	19.8%	5.4%	8.1%	16.74%	4.13%	6.73%	-3.1%	-1.2%	-1.4%
Separated / divorced / widowed	20.0%	30.8%	19.5%	19.07%	28.24%	18.33%	-0.9%	-2.5%	-1.2%
Race/ethnicity									
White, non-Hispanic	73.3%	79.8%	84.2%	73.68%	81.51%	83.64%	0.4%	1.7%	-0.6%
Black, non-Hispanic	11.4%	9.4%	9.8%	9.69%	7.44%	4.84%	-1.7%	-2.0%	-5.0%
Other	4.7%	3.6%	1.9%	6.68%	5.08%	6.73%	2.0%	1.5%	4.8%
Hispanic	10.6%	7.2%	4.1%	9.96%	5.97%	4.80%	-0.7%	-1.2%	0.7%
Education									
Less than HS	15.5%	21.2%	11.0%	13.70%	15.36%	3.12%	-1.8%	-5.9%	-7.8%
HS or equivalent	32.3%	33.8%	34.8%	28.86%	32.90%	20.54%	-3.4%	-0.9%	-14.2%
Some college	26.8%	22.5%	31.0%	31.61%	26.00%	46.06%	4.8%	3.6%	15.0%
Bachelor degree +	25.4%	22.6%	23.3%	25.83%	25.74%	30.28%	0.4%	3.2%	7.0%
Household income									
<$10,000	7.2%	8.4%	4.4%	5.90%	5.70%	2.29%	-1.3%	-2.7%	-2.1%
$10,000 - $24,999	18.8%	23.8%	18.6%	16.48%	18.61%	10.83%	-2.3%	-5.2%	-7.8%
$25,000 - $49,999	29.3%	29.0%	33.1%	36.74%	36.12%	37.81%	7.5%	7.1%	4.7%
$50,000 - $74,999	20.3%	17.2%	21.8%	23.19%	20.56%	27.24%	2.9%	3.4%	5.5%
$75000+	24.5%	21.5%	22.1%	17.68%	19.01%	21.83%	-6.8%	-2.5%	-0.3%
Region									
Northeast	19.2%	20.1%	17.4%	18.84%	19.10%	13.02%	-0.3%	-1.0%	-4.4%
Midwest	22.8%	23.0%	22.9%	26.69%	27.19%	34.92%	3.9%	4.2%	12.0%
South	35.6%	36.3%	38.3%	34.54%	37.73%	31.12%	-1.1%	1.5%	-7.2%
West	22.5%	20.7%	21.3%	19.93%	15.98%	20.95%	-2.5%	-4.7%	-0.4%
Average of absolute value of deviations							2.3%	2.5%	4.3%

Table A2b: Frequencies of CPS December 2001 compared to our weighted KN survey data (fielded December 2001-January 2002)

	CPS December 2001			KN Weighted Distributions of Completes			Differences KN Weighted - CPS		
	Age 21+	Age 50+	Veterans 21+	Age 21+	Age 50+	Veteran s 21+	Age 21+	Age 50+	Veterans 21+
Gender	Age 21+								
Male	47.8%	45.8%	94.4%	46.2%	41.0%	95.3%	-1.6%	-4.8%	0.9%
Female	52.2%	54.2%	5.6%	53.8%	59.0%	4.7%	1.6%	4.8%	-0.9%
Age range									
21-24	7.9%	0.0%	1.1%	5.7%	0.0%	0.5%	-2.2%	0.0%	-0.6%
25-34	19.1%	0.0%	7.1%	17.8%	0.0%	6.6%	-1.3%	0.0%	-0.5%
35-44	22.9%	0.0%	12.3%	23.1%	0.0%	13.4%	0.2%	0.0%	1.1%
45-54	20.1%	23.8%	20.9%	21.1%	22.2%	22.2%	1.0%	-1.6%	1.3%
55-64	12.9%	32.7%	20.8%	15.7%	37.9%	22.6%	2.9%	5.2%	1.8%
>65	17.1%	43.6%	37.8%	16.5%	39.9%	34.7%	-0.6%	-3.7%	-3.1%
Marital status									
Married	60.2%	63.9%	72.4%	63.0%	67.1%	76.1%	2.8%	3.3%	3.7%
Single	19.8%	5.4%	8.1%	16.6%	4.0%	6.5%	-3.2%	-1.4%	-1.6%
Separated / divorced / widowed	20.0%	30.8%	19.5%	20.4%	28.9%	17.4%	0.4%	-1.9%	-2.1%
Race/ethnicity									
White, non-Hispanic	73.3%	79.8%	84.2%	73.3%	82.0%	82.2%	0.0%	2.2%	-2.0%
Black, non-Hispanic	11.4%	9.4%	9.8%	11.3%	9.8%	8.9%	-0.1%	0.4%	-0.9%
Other	4.7%	3.6%	1.9%	3.4%	2.4%	2.9%	-1.3%	-1.2%	1.0%
Hispanic	10.6%	7.2%	4.1%	12.1%	5.8%	6.0%	1.5%	-1.4%	1.9%
Education									
Less than HS	15.5%	21.2%	11.0%	24.6%	29.7%	11.0%	9.1%	8.5%	0.0%
HS or equivalent	32.3%	33.8%	34.8%	33.3%	34.8%	32.8%	1.0%	1.0%	-2.0%
Some college	26.8%	22.5%	31.0%	22.1%	17.5%	30.6%	-4.7%	-5.0%	-0.4%
Bachelor degree +	25.4%	22.6%	23.3%	20.0%	18.1%	25.5%	-5.4%	-4.5%	2.3%
Household income									
<$10,000	7.2%	8.4%	4.4%	8.4%	8.3%	3.7%	1.2%	-0.1%	-0.7%
$10,000 - $24,999	18.8%	23.8%	18.6%	19.9%	20.8%	12.4%	1.2%	-3.0%	-6.2%
$25,000 - $49,999	29.3%	29.0%	33.1%	37.7%	38.3%	41.1%	8.5%	9.3%	8.0%
$50,000 - $74,999	20.3%	17.2%	21.8%	20.2%	19.0%	25.0%	-0.1%	1.8%	3.2%
$75000+	24.5%	21.5%	22.1%	13.9%	13.7%	18.1%	-10.6%	-7.8%	-4.0%
Region									
Northeast	19.2%	20.1%	17.4%	19.2%	21.4%	17.5%	0.0%	1.3%	0.1%
Midwest	22.8%	23.0%	22.9%	22.8%	22.1%	21.9%	0.0%	-0.9%	-1.0%
South	35.6%	36.3%	38.3%	35.6%	38.0%	36.6%	0.0%	1.8%	-1.7%
West	22.5%	20.7%	21.3%	22.5%	18.6%	24.1%	0.0%	-2.1%	2.8%
Average of absolute value of deviations							2.2%	2.8%	2.0%

Table A3: Comparison of KN Panel and NHIS Data on Health Measures

Measure	KN (%)	NHIS (%)	Difference (in % points)
Ever Smoke	44.8	45.5	-0.7
Current Smoke	24.7	23.3	1.4
Diabetes	7.1	6.7	0.4
Ulcer	7.1	7.3	-0.2
Migraine	12.2	14.9	-2.7
Stroke	1.8	2.2	-0.4

Table A4: Comparison of Condition Prevalence in the Stanford survey and the 2000 NHIS

Topic	Survey	Question Structure	N	%
Hypertension	Stanford	"Has a doctor or other health care provider ever told you that you have high blood pressure or hypertension?"	8930	29%
	NHIS	Have you ever been told by a doctor or other health professional that you had hypertension, also called high blood pressure?"	31017	24%
Heart Problems	Stanford	"Has a doctor or other health care provider ever told you that you have had a heart attack, or have coronary heart disease, angina, heart failure, or other heart problems?"	8917	11%
	NHIS	Four separate questions: "Have you ever been told by a doctor or other health professional that you had…" "coronary heart disease?", "angina, also called angina pectoris?", "a heart attack (also called myocardial infarction)?", "any kind of heart condition or heart disease (other than the ones I just asked about)?" A "yes" answer to any of the four defines a "yes" for the category heart problems.	31014	11%
Cancer	Stanford	"Has a doctor or other health care provider ever told you that you have cancer?"	8914	6%
	NHIS	"Have you ever been told by a doctor or other health professional that you had cancer or a malignancy of any kind?"	31029	7%
Diabetes	Stanford	"Has a doctor or other health care provider ever told you that you have diabetes or high blood sugar" with response options "yes," "no," and "borderline." Figure given counts "yes" or "borderline"	8912	12%
	NHIS	"[if female, "other than during pregnancy,"] Have you ever been told by a doctor or health professional that you have diabetes or sugar diabetes" with response options "yes," "no," and "borderline" Figure given counts "yes" or "borderline"	31030	8%

Table A5: Comparison of visit utilization measures in the Stanford survey and the 2000 NHIS

			N	None	1	2-5	6+
Provider Office Visits in Last Year	Stanford	In the past year, how many times did you see a medical doctor, physician assistant, nurse, or nurse practitioner in a doctor's office or medical clinic?" with response categories "None," "1," "2-5," "6-10," 11-20," and "more than 20"	8903	16%	16%	44%	24%
	NHIS	"During the past 12 months, how many times have you seen a doctor or other health care professional about your own health at a doctor's office, a clinic, or some other place? Do not include times you were hospitalized overnight, visits to hospital emergency rooms, home visits, dental visits, or telephone calls" with response categories "None," "1," "2-3," "4-5," "6-7," "8-9," "10-12," "13-15," "16 or more"	30569	19%	17%	39%	25%
ER Visits in Last Year	Stanford	"In the last year, how many times did you see a medical doctor, physician assistant, nurse, or nurse practitioner in an emergency room?" with response categories "None," "1," "2-5," "6-10," "11-20," and "more than 20"	8718	72%	16%	10%	2%
	NHIS	During the past 12 months, how many times have you gone to a hospital emergency room about your own health? (this includes emergency room visits that resulted in a hospital admission)" with response categories "None," "1," "2-3," "4-5," "6-7," "8-9," "10-12," "13-15," "16 or more"	30746	80%	13%	6%	1%

Table A6: Comparison of visit utilization measures in the Stanford survey and the 1999 CTSHS

			N	None	1	2+
Provider Office Visits in Last Year	Stanford	In the past year, how many times did you see a medical doctor, physician assistant, nurse, or nurse practitioner in a doctor's office or medical clinic?" with response categories "None," "1," "2-5," "6-10," 11-20," and "more than 20"	8903	16%	16%	68%
	CTSHS	Two questions: "Since {date 12 months ago}, about how many times has [fill name] seen a doctor? Do not count doctor seen while an overnight patient in a hospital or in the emergency room" with coded responses continuous for 0-19, and then 20 or more." and "How many times has [fill name] seen a nurse practitioner, physician's assistant [or midwife] during the past 12 months?" with continuous responses 0-3, and then 4 or more	30831	19%	15%	66%
ER Visits in Past Year	Stanford	"In the last year, how many times did you see a medical doctor, physician assistant, nurse, or nurse practitioner in an emergency room?" with response categories "None," "1," "2-5," "6-10," "11-20," and "more than 20"	8718	72%	16%	12%
	CTSHS	"During the past 12 months, how many time has [fill name] gone to a hospital emergency room?" [note that answers were also adjusted for a hospital admissions variable to include ER visits that led to an admission]. The coded responses are 0, 1, 2, 3, 4, 5 or more.	30831	76%	16%	9%

Table A7: Comparison of hospitalization measures in Stanford survey, 2000 NHIS, and 1999 CTSHS

		N	None	1	2	3+
Stanford	"In the last year, how many times have you been a patient in a hospital where you stayed at least one night?" with response categories "None," "1," "2," and "3 or more"	8910	87%	9%	2%	2%
NHIS	Two questions: "During the past 12 months was {person} a patient in a hospital overnight? (Do not include an overnight stay in the emergency room)." and "How many different times did {person} stay in any hospital overnight or longer during the past 12 months?"	37427	90%	7%	2%	1%
CTSHS	"How many different times did [fill name] stay in any hospital overnight or longer during the past 12 months?"	30831	88%	9%	2%	1%
